# Recent Advances in Genetic Predisposition of Myasthenia Gravis

**DOI:** 10.1155/2013/404053

**Published:** 2013-11-05

**Authors:** Zoi Zagoriti, Manousos E. Kambouris, George P. Patrinos, Socrates J. Tzartos, Konstantinos Poulas

**Affiliations:** ^1^Laboratory of Molecular Biology and Immunology, Department of Pharmacy, School of Health Sciences, University of Patras, 26504 Rio, Patras, Greece; ^2^Department of Biochemistry, Hellenic Pasteur Institute, 127 Vas. Sofias Avenue, 11521 Athens, Greece

## Abstract

Myasthenia gravis (MG) is an autoimmune disease mediated by the presence of autoantibodies that bind to components of the neuromuscular junction, causing the symptoms of muscular weakness and fatigability. Like most autoimmune disorders, MG is a multifactorial, noninherited disease, though with an established genetic constituent. The heterogeneity observed in MG perplexes genetic analysis even more, as it occurs in various levels, including diverse autoantigens, thymus histopathology, and age at onset. In this context of distinct subgroups, a plethora of association studies, discussed in this review, have assessed the involvement of various HLA and non-HLA related loci in MG susceptibility, over the past five years. As expected, certain HLA alleles were strongly associated with MG. Many of the non-HLA genes, such as *PTPN22* and *CTLA-4*, have been previously studied in MG and other autoimmune diseases and their association with MG has been reevaluated in more cohesive groups of patients. Moreover, novel risk or protective loci have been revealed, as in the case of *TNIP1* and *FOXP3*. Although the majority of these results have been derived from candidate gene studies, the focal point of all recent genetic studies is the first genome-wide association study (GWAS) conducted on early-onset MG patients.

## 1. Introduction

Myasthenia gravis (MG) is an autoimmune disease affecting components of the neuromuscular junction and thus disrupting the signal transduction over the postsynaptic membrane [1]. It is a heterogeneous disease, both clinically and biologically, delineated by the presence of specific autoantibodies which are the main pathogenic and diagnostic feature.

MG is well-characterized at the effector stage with three autoantigens accounting for nearly 90–95% of the clinical cases; the major target, in 80–85% of MG patients, is the muscle acetylcholine receptor (AChR) [2], whereas in the rest of the MG patients, pathogenic autoantibodies are directed towards the muscle-specific tyrosine kinase (MuSK) [3] or the low-density lipoprotein receptor-related protein 4 (LRP4) [4, 5]. However, the presence of only three targets does not mean that there are only three epitopes and three autoantibody idiotypes. Actually, the designation of an extracellular region of the AChR alpha-subunit as MIR—Main Immunogenic Region—clearly implies a multitude of possible epitopes [6, 7]. It is noteworthy that even a single patient might have autoantibodies against more than one epitopes [7].

Even more heterogeneity can be observed among patients; the age at onset which, usually, delineates the groups of early-onset MG (EOMG) < 40 years and late-onset MG (LOMG) > 40 years is strongly related to the gender and constitutes a crucial subdivision of the MG population [8]. Another cause of heterogeneity is the thymus histopathology, referring to thymoma or thymus hyperplasia, which seems to “shadow” genetic analysis, as it does not fall within the EOMG/LOMG classification used in most association studies. In general, the EOMG subset is characterized by female predominance (~2-3 : 1 female to male ratio) and, usually, by a hyperplastic thymus, whereas LOMG shows a male bias, with normal or atrophic thymus, in most of the cases [8]. The presence of anti-titin antibodies (ATA), although of no apparent active role in MG, is thought to signify a more homogeneous group of LOMG patients [9]. Clinically, the extent and the severity of the symptoms, the progression rate (ocular versus generalized type of MG), and the response to different therapeutic approaches complicate the issue even further [10].

## 2. Genetic Approaches in Autoimmune Disorders

In contrast to Congenital Myasthenic Syndromes (CMS) which, usually, follow Mendelian principles of heredity and do not entail an autoimmune component, MG is a noninherited disease and like most autoimmune disorders, it is considered to have a multifactorial underlying basis. In fact, it is assumed that the pathogenesis of autoimmune diseases is caused by a complex interaction between multiple genotypes of low penetrance and environmental factors, including pathogen exposure, particularly Epstein-Barr virus [11–13], sex hormones [14], and lifestyle habits, such as cigarette smoking [15]. Twin studies reviewed in [16] revealed that high concordance rates among monozygotic compared to dizygotic twins, observed in several autoimmune diseases including MG, are suggestive of a strong genetic component. However, because of epistasis, the individual effect of a gene may be masked or altered through its interaction with other genes. Moreover, the genetic element seems to be restrained and complicated, as the disease-associated alleles of risk loci appear in healthy individuals, as well.

Over the past decade, whole genome resequencing, along with the rapid evolution of high throughput technologies and statistical tools have provided novel insights in genetic studies. The development of a new prospect in genetics, genome-wide association studies (GWAS), was crucial for the identification of numerous loci, involved in autoimmune diseases.

The vast majority of genetic factors that have been implicated in autoimmune disease susceptibility are common variants, predominantly, single nucleotide polymorphisms (SNPs) rather than insertion/deletion polymorphisms or microsatellites. SNPs are found quite frequently throughout the human genome, while their impact on gene expression or protein's function is not always very noticeable.

A genetic study has been traditionally implemented by either linkage analysis or association analysis. The latter is, nowadays, further diversified into candidate gene association study and GWAS. Linkage analysis allows inclusion of vastly different subjects, as it focuses on transmission patterns and not on the residual picture. However, it needs well-established pedigrees, which is not an easy task in general, and the low rate of MG familial recurrence makes it even more difficult to construct. On the other hand, candidate gene association study allows inclusion of completely unrelated subjects, as it ignores transmission patterns, but it requires extreme caution in grouping the test population in relevantly cohesive groups in order to unveil association between genetic loci and the syndrome under study. Also, there must be some *a priori* basis for suspecting that the candidate gene may be correlated with the disease.

Being a combination of the former two approaches, the Transmission-Disequilibrium Test (TDT) allows using the base of case-control association studies to determine linkage, through marker transmission in rather shallow family environments (i.e., with data collected or projected for as little as two generations and sometimes with some links missing) [17]. The GWAS is a step beyond, as it is not the correlation with a certain, candidate locus that must be established or overruled, but any association with any of the genomic markers, irrespectively of physical distance, is sought for. Such an endeavor is possible thanks to the existence and the considerable extent of linkage disequilibrium (LD), which ensures monoblock transmission, through successive generations, of long chromosomal parts, with polymorphic alleles located on them being “in phase”; meaning that, with novel mutagenesis excluded, they are transmitted with identical allelic status forming a haplotype. Thus, instead of millions of markers, one has just to check one marker per LD block, at least in theory.

Convenient for detecting candidate loci without previous assumption as it may LD readily lowers the discrimination potential of an association study, and hence, it impairs the accurate location of the implicated site. High-throughput analysis alleviates this by increasing the number of markers interrogated to more informative and discriminative levels, once appropriate depth of analysis is ensured by assembling adequate sample sizes. All three genetic approaches establish, by definition, statistical correlations between candidate disease loci and phenotypes, but they do not produce causative relationship, especially in multifactorial diseases and syndromes, where a combination of loci may produce an end result unattainable by any of the constituents.

In this review, we attempted to incorporate all the recent data of the international literature regarding the genetic associations of MG, generated mostly from candidate gene studies, but, also, from the first GWAS conducted on MG patients. We focused mainly on studies published over the past five years, as previous studies—before 2008—have been covered in a review by Giraud and coworkers [18].

## 3. Data Derived from the First GWAS in EOMG Patients

In 2012, the first GWAS for MG [19] was conducted through an international collaboration of multiple centers that led to the recruitment of 740 MG cases. After applying strict quality criteria to obtain the appropriate clustering of the samples, the 649 patients, who were, finally, included in the study, were of North European descent and developed the disease early, with age at onset >10 years and <40 years, as they comprise a more homogeneous group, suitable for genetic analysis. In terms of thymic histological condition, MG patients were diagnosed with thymus hyperplasia, whereas a strong female bias (82.9% of the overall MG group) was noticed. As probably expected, the study detected the strongest association in the Human Leukocyte Antigen (HLA) Class I locus (rs7750641) which, after imputation and conditional analyses of the broad HLA region, led to the determination of HLA-B*08 allele as the main risk allele (Table 1). A higher degree of association between the HLA-B*08 allele and EOMG was revealed when considering only the women. Regarding the HLA-DRB1*0301 allele that had been previously associated with thymus hyperplasia-related MG [9], the modest signal observed in the unconditioned analysis was clearly reduced when conditioning on HLA-B8. However, two other alleles of the HLA Class II region, DRB1*16 and DRB1*0701, conferred a positive and negative association, respectively, that reached statistically significant levels (Table 1), confirming previous findings [9]. This GWAS, also, verified the association between MG with thymus hyperplasia and the rs2476601 SNP in the *PTPN22* locus (discussed below) which has been reported in previous studies [20, 21]. In addition, a novel association was established with TNFAIP3-interacting protein 1 (TNIP1) involved in the negative regulation of NF-*κΒ* [19]. Applying imputation around the *TNIP1* locus, a strong association was demonstrated with the rs2233290 SNP, causing a proline to alanine substitution at position 151 of the coding region [19]. The fact that this variant does not lie within any of the functional domains of the protein possibly explains the lack of association with TNFAIP3, which was, also, observed in a candidate gene study of Hellenic MG population [22].

## 4. HLA Loci Susceptible to MG Occurrence

In the 1970s, the HLA was the first identified genetic region with a significant role in predisposition of many autoimmune diseases. However, because of the large number of highly polymorphic genes located at this region and related to the immune function and because of the strong linkage disequilibrium across HLA, the identification of the precise alleles associated with disease susceptibility remains challenging.

### 4.1. Thymoma-Related MG

Thymomas are neoplasms of thymic epithelial cells, with ~30–45% of them associated with MG. Except for the increased MG prevalence, many other autoimmune diseases, also, cooccur in patients with tumours of the thymus [33]. Histologically, thymomas are considered to be heterogeneous tumours. According to the World Health Organization (WHO), thymomas are classified into five histological types, differentiated by their neoplastic (epithelial) and nonneoplastic (lymphocytes) component proportions [34]. These are types A, AB, B1, B2, and B3, in order of increasing malignancy, and they have all been observed in MG patients [35].

MG patients with thymoma comprise a distinct subset of the disease, both pathologically and genetically. No reproducible HLA association has been reported in MG with thymoma.

In 2001, a case-control study conducted by Garchon and coworkers revealed no significant association of Class II HLA-DRB1 locus in 106 French MG patients with thymoma [36]. However, histological data about thymoma subtypes were not available which may partially explain the lack of association.

A subsequent study by the same research group, comprising 78 French MG patients with thymoma, investigated the effect of Class I HLA-A locus to the occurrence of paraneoplastic MG. An increased frequency of HLA-A*25 allele was found in the whole group of patients (Table 1). Considering only the subgroup of 27 MG patients with a B2 type thymoma, the analysis demonstrated a negative association of the HLA-A*02 allele (Table 1), indicating a potential protective role to the development of B2 thymoma [23].

Recently, the Class II HLA-DQA1 and DQB1 genes were associated with thymoma-related MG in an Asian population. Among the 102 northern Chinese MG patients who constituted a very heterogeneous study group, 41 presented with thymoma, with B2 type being the most prevalent histological subgroup (51%). A significant increase of HLA-DQA1*0401 and DQB1*0604 alleles was disclosed in MG patients with thymoma (Table 1), compared to the ones without thymoma and to the control group [24]. 

Finally, no association was observed in the case of 30 Norwegian MG patients with thymoma who were genotyped for HLA Class I and II loci [26].

### 4.2. Nonthymomatous MG

The involvement of HLA-DRB1 gene in MG occurrence—under the perspective of its heterogeneity—was assessed through a large study including 656 sporadic MG cases of Caucasian origin. The group of 192 MG patients with thymus hyperplasia presented a significantly increased frequency of the DR3 allele and a concurrent decrease of the DR7 allele frequency, compared to the 10,235 healthy subjects (Table 1) [9]. Moreover, through a relative predispositional effect (RPE) analysis of DR alleles, the DR16 and DR9 alleles, sequentially according to their strength, were shown to be associated with susceptibility to MG and thymus hyperplasia (Table 1) [9]. In order to achieve a greater level of homogeneity, the MG patients not being detected with any thymus anomalies, were categorized by the presence of ATA. The 59 ATA-positive patients appeared to be associated with the DR7 allele, whilst a negative association was observed in the case of the DR3 allele (Table 1), revealing a reverse outcome from the one noticed in MG patients with thymus hyperplasia and in ATA-negative patients who were associated with DR3 (Table 1), as well [9]. Additionally, this study investigated the transmission of the DR3 allele, using the transmission/disequilibrium test in 56 families that consisted of one offspring with MG and thymus hyperplasia and one or two heterozygous parents. This family-based association test determined the presence of genetic linkage between the HLA-DR3 allele and MG with thymus hyperplasia [9].

In the sequel study, Garchon and coworkers reevaluated the contribution of the extended 8.1 HLA haplotype, including Class I HLA-A1 and B8 loci and Class II HLA-DR3 allele, to the occurrence of MG with thymus hyperplasia. Overall, 27 markers, most of them microsatellites, covering the entire region of HLA complex, were genotyped. The family-based transmission disequilibrium test proved the distorted transmission of a core part of the 8.1 haplotype in 46 cases. This 1.2 Mb segment, mapped in the central region of the 8.1 haplotype between the BAT3 and C3-2-11 markers, encompasses the Class III and proximal Class I loci and constitutes the actual causative locus, termed MYAS1 [37]. Furthermore, a quantitative trait locus associated with anti-AChR autoantibody production is suggested to be situated within the MYAS1 [37]. Finally, in the context of a case-control association study, it was supported that the risk conferred by the 8.1 haplotype probably corresponds to an additive genetic model [37].

More recent data derived from an association study of 1,472 SNPs covering the overall MHC region, in a cohort of 438 MG cases from Sweden, determined the Class I HLA locus as the most susceptible in MG [25]. More specifically, the strongest association was detected in the case of rs2523674 SNP mapped 3.5 kb downstream of the HLA complex protein 5 *(HCP5)* gene, while the imputed HLA-C*0701 allele was also associated, in an independent way (Table 1) [25]. Although this study scrutinized a large set of genetic variants across the vast MHC region, it disregarded the heterogeneous clinical and biological profiles of the disease that could lead to distinct HLA association signals.

In order to assess this heterogeneity, a case-control association study in the Norwegian population examined the HLA-A, -B, -C, and  -DRB1 loci in well-defined subgroups of MG patients sorted by the age at onset (Early-onset < 40 years, Late-onset > 60 years and intermediate group = 41–59 years) and the histology of the thymus (thymoma or nonthymoma). The greatest association of LOMG (>60 years as determined in this study) was with the DRB1*15 : 01 allele (Table 1), while the DR7 allele showed a modest association which ceased to be significant in the case of MG patients with ATA and age at onset over the 40 years [26]. In the group of EOMG patients, the previously demonstrated association with the HLA-A*01, -B*08, -C*07, and  -DRB1*0301 alleles was, also, confirmed in this study and further conditional analysis revealed that HLA-B*08 was the responsible allele for the strongest risk to MG susceptibility, detected for this haplotype (Table 1) [26]. A negative association with the DRB1*13 : 01 allele was observed in both EOMG and LOMG patients (Table 1) suggesting its protective role in MG [26].

Another study of HLA-DRB1 locus, in a northern Han Chinese cohort of MG patients, showed a significantly increased frequency of DRB1*09 allele in EOMG patients and a corresponding raise of DRB1*07 allele frequency in LOMG (Table 1) [27]. The DRB1*09 was also associated with the negative to anti-AChR antibodies subgroup, but the strongest association of this allele was uncovered in the case of MG patients with the ocular type of the disease (Table 1) [27]. It is worth mentioning that unlike Caucasian populations, no statistically significant differences were observed in the frequency of DRB1*03 allele in this study.

In Saudi MG patients, DNA typing of HLA Class I and II loci revealed a robust association only with the B*08 allele, detected mostly in the female and early-onset disease part of the study group [28]. In addition, HLA-B*08 and -DRB1*03 alleles were shown to be in linkage disequilibrium, thus, forming a haplotype from which the A1 locus appeared to be unexpectedly excluded [28]. The HLA-DQB1 locus did not show any correlation with MG in Saudi patients, but in a study encompassing Iranian patients, HLA-DQA1*0101/2 and DQB1*0502 alleles were positively associated with MG (Table 1) [29].

The contribution of HLA-DQ and DR loci to MG predisposition was, also, assessed in 48 Tunisian patients with generalized MG and almost half of them being seronegative to anti-AChR antibodies (20/48). The DRB1*03 and DQB1*02 alleles were significantly more prevalent in the EOMG patients, while the LOMG patients were associated with the DRB1*04 and DQB1*0302 alleles (Table 1) [30]. When comparing the allele frequencies according to gender, the results were the opposite of what would be expected, as the EOMG-associated DRB1*03 and DQB1*02 alleles presented in a significantly increased frequency in male patients and the DRB1*04 and DQB1*0302 alleles in women (Table 1) [30]. This contradiction could be in part due to the limited number of MG patients participating in the study.

### 4.3. MuSK-MG

MuSK-MG is a separate clinical entity with discrete clinical features and patterns of weakness, compared to AChR-MG. In MuSK-MG, no abnormalities have been reported in the histology of the thymus [38].

The HLA-DR14-DQ5 haplotype was strongly associated with MuSK-MG in 23 white Dutch patients (Table 1), while the B8-DR3 haplotype, which has been consistently associated with early-onset AChR-MG, showed no significant difference [31].

Another study comprising 37 Italian MuSK-MG patients demonstrated strong association with DQB1*0502 and DRB1*16 alleles (Table 1) [32]. Since the molecular DQB1*0502 allele corresponds to the serologic DQ5, these results confirm the association of MuSK-MG with DQ5 established by the above-mentioned study.

All statistically significant HLA associations with MG, generated from the above-mentioned studies, are summarized in Table 1.

## 5. Non-HLA Related Loci Associated with MG Predisposition

### 5.1. Protein Tyrosine Phosphatase Nonreceptor Type 22 (PTPN22): A General Autoimmunity Risk Factor

*PTPN22* located on 1p13.3–p13.1 chromosomal region encodes for a cytoplasmic tyrosine phosphatase which is specifically expressed in lymphoid cells. Structurally, PTPN22 consists of two functional domains: the N-terminal catalytic-dephosphorylation domain and the C-terminal binding domain that mediates its interaction with the SH3 domain of the intracellular C-src tyrosine kinase (CSK) [39]. Through the complex with CSK, PTPN22 functions as a molecule-regulator of the TCR signalling pathway, leading to the suppression of T-cell response [40]. The missense SNP rs2476601 (1858C>T), which leads to an arginine to tryptophan substitution (R620W), has been reproducibly associated with multiple autoimmune diseases, MG included, as reviewed in [41]. As a matter of fact, a meta-analysis, conducted recently on the association of rs2476601 with a plethora of autoimmune diseases, established the correlation of this variant with rheumatoid arthritis, MG, lupus, type I diabetes, and several other diseases, but not with a cluster of diseases affecting the skin, the gastrointestinal tract, or immune privileged areas, such as psoriasis, Crohn's disease, multiple sclerosis, and so forth [42]. Recent data derived from the study by Zhang et al. indicated an impairment of 620W variant stability, in the protein level, caused by an increased degradation rate compared to the wild-type 620R protein [43].

Following the study of the French MG population [21], recent data have confirmed the correlation of R620W to MG susceptibility in other Northern European populations, as well. A case-control study in a large cohort of Swedish Caucasian MG patients revealed that the minor T allele showed the greatest association with the subgroup of MG with thymus hyperplasia and anti-AChR antibodies (Table 2) [20]. The same study evidenced a significant increase of IL-2 producing cells, after stimulation of PBMC with the human AChR protein, in patients with the T allele. This observation is inconsistent with the results of decreased IL-2 levels, demonstrated by Vang and coworkers, in patients with type I autoimmune diabetes carrying the 620W allele [44].

An association study of the R620W variant in Hungarian and German MG patients proved that the *PTPN22* 1858T allele was associated with MG predisposition, only, in the subgroup of nonthymoma patients with detectable ATA (Table 2) [45], leading to conflicting conclusions compared to the French MG study, in which the association was established in the exactly opposite case of non-thymoma patients lacking ATA [21].

Another study in German Caucasian MG patients was the first to uncover a strong association between +1858T genotypes and thymoma-related MG (Table 2) [46]. A statistically significant association was reported with EOMG subjects, too (Table 2). It is worth mentioning that the intratumorous IL-2 expression levels were found to be reduced in patients bearing the +1858T allele [46].

In discordance with the above studies, determination of rs2476601 allele frequencies in a large Italian group of MG patients showed no statistically significant differences between patients and healthy controls [47]. Instead, the rs2488457 SNP which resides in the *PTPN22* promoter region (-1123G>C) appeared to be associated with MG characterized by low titer anti-AChR antibodies (Table 2) [47]. This absence of association between MG predisposition and +1858T allele might be in accordance with the decreased allele frequency observed in the general population of the southern European countries, compared to the northern and eastern part of the continent [48].

A meta-analysis of the four above studies performed on allele frequencies of the *PTPN22* rs2476601 verified the association of the T allele with nonthymomatous MG [47]. The statistical power of association was significantly reduced when the results from the Italian group were added to the meta-analysis data [47].

An overview of the results derived from the genetic association studies for *PTPN22* is presented in Table 2.

### 5.2. Association of Other Non-HLA Loci with MG Susceptibility

To begin with, one of the most important associations is that of the rs16862847 SNP located at the promoter region of the *CHRNA1*—encoding the alpha-subunit of the muscle AChR pentameric channel—which has been constantly associated with MG, as discussed in detail in [18].

The (cytotoxic T lymphocyte-associated 4) CTLA-4/CD152 membrane receptor exerts its impact as suppressor of activated T-cells via binding to B7 costimulatory molecules on antigen-presenting cells. In the absence of a trigger, nonstimulated peripheral T lymphocytes express an alternative transcript which encodes a soluble form of CD152 (sCD152) [49]. In addition to previous studies reporting a probable association between the +49A/G coding variant and the subset of thymoma-related MG [50, 51], the involvement of SNPs located at the promoter region of *CTLA-4* has also been evaluated. Two SNPs, at positions -1772T/C and -1661A/G upstream of *CTLA-4,* were shown to be associated with MG in a group of 165 MG patients of Swedish origin [52]. Because of their location within regulatory elements—the NF-1 and c/EBP binding sites—these polymorphisms plausibly have an effect on gene transcription or even splicing, as implied by the increased levels of sCD152, in -1772T/C heterozygote MG patients [52].

Given the indisputable role of glycobiology in immunity and inflammation, galectins—members of the glycan-binding protein family—were considered as good candidates in the investigation of genetic factors predisposing to autoimmunity. Galectins are soluble proteins containing a carbohydrate recognition domain with high affinity to N-acetyllactosamine oligosaccharides [53]. They mediate a broad spectrum of immunomodulatory activities both intracellular and extracellular, including induction of apoptosis, negative regulation of T-cell response, cell adhesion, and pre-mRNA splicing [54]. The rs2737713 polymorphism which leads to a phenylalanine to tyrosine substitution (F19Y) in the *LGALS8* locus, encoding galectin-8, appeared to be moderately associated with MG [55]. A previous study of the same research group examined two SNPs in the 5′ upstream sequence of the galectin-1 gene (*LGALS1)*, along with two SNPs in regulatory regions of the *interleukin receptor 2*β** gene (*IL2R*β**) which resides in the 22q13 chromosomal region, too. A strong association was identified between MG and the haplotype formed by SNPs rs4820293 and rs743777 of *LGALS1* and *IL2R*β**, respectively, but functional studies did not verify an impact of rs4820293-different genotypes on gene expression [56].

Another cytokine receptor, this time interleukin-4 receptor alpha (IL4R*α*), was examined in a cohort of Hungarian MG patients recruited from the NEPSYBANK (Hungarian Neurological and Psychiatric Biobank), [57], as occurred in the two above-mentioned studies. In this survey, three missense variants in the *IL4R*α** (I75V, S503P, and Q576R) which are thought to disrupt the signal transduction of interleukin-4 (IL-4) were selected. A statistically significant association was observed only in the case of I75V polymorphism and, particularly, when the 75V allele existed in homozygosity [58].

Interleukin-10 (IL-10) is an anti-inflammatory cytokine which exerts its function by downregulating T_H_1-mediated responses, whereas it constitutes a proliferation and differentiation factor for activated B cells [59]. Three SNPs, at positions -1082 (A/G), -819 (T/C), and -592 (A/C) in the 5′ flanking sequence of *IL-10* determine the formation of three haplotypes (GCC, ACC, and ATA) that are implicated in IL-10 production levels, *in vitro *[60]. A study conducted on the Norwegian population proved the association of the low expression-related genotypes, ACC/ACC and ATA/ATA, with the subgroups of ATA-positive and EOMG patients, respectively [61]. However, in the Hellenic population, the GCC/GCC genotype conferring for high IL-10 levels showed a statistical trend of association when the EOMG and LOMG subsets were compared [22].

Foxp3 is a transcription factor, member of the forkhead/winged-helix family, with a specific role in the function of CD4^+^/CD25^+^ T regulatory cells. A marked reduction in T_reg_ immunosuppressive activity *in vitro* has been identified in MG patients, accompanied by a decrease in *FOXP3* expression [62]. A recent study in Han Chinese MG cases attempted to assess the contribution of two SNPs, rs3761548 (−3279A/C) and rs2280883 (IVS9+459A/G), in the *FOXP3* gene to MG susceptibility. Statistically significant differences in the genotype distribution of the intronic variant were observed between patients and controls, uncovering a potential protective role of the IVS9+459G allele to the development of MG [63]. No association was evidenced in the case of rs3761548, even after stratification of the subjects according to age at onset, gender, histology of the thymus, and clinical classification [63].

## 6. Genetic Loci Not Associated with MG

The studies resulting in no statistically significant association are minimally published. This fact, although unconstructive, as it denies clues for more promising direction of further research, might be a justified policy, since better selected population groups and enhanced correlation protocols might lead to positive future findings. Briefly, in the last five years, *Interferon Regulatory Factor 5 *(*IRF5*) [22], *TNF*α*-induced protein 3 *(*TNFAIP3*) [22],* IL10* [22]*, Stromal Cell Derived Factor-1 *(*SDF1*) [64]*, Programmed Death-1 *(*PD-1*) [65]*, Oestrogen Receptor Alpha *(*OR*α**) [66], *Class II Transactivator *(*CIITA*) [67], and *Protein Tyrosine Phosphatase, Receptor type C* (*PTPRC*), also designated as CD45 [68], have been tested and temporarily rejected. The list of nonassociated loci becomes more extensive if previous studies discussed elsewhere [18] are taken into account. 

## 7. Conclusion

The purely autoimmune nature of MG entails the fact that complex pathogenetic mechanisms cause the deregulation of normal immune function, so that an immune response targeting the MG-related autoantigens would develop. As it was expected, numerous studies have verified quite strong associations with various HLA alleles, depending largely on the traits of the study group. Moreover, a series of non-HLA loci were shown to be implicated, enriching our understanding of the multifaceted autoimmune procedures and thus unraveling the specific role of these genes in immunological pathways. Most of these risk loci have been repeatedly associated with several autoimmune diseases, indicating the existence of common underlying pathogenesis in autoimmunity. Thus, MG-associated loci might exert their implication through upregulation of the immune response, inhibition of immunosuppressive mechanisms, or impairment of the delicate procedure of regulating the discrimination between autologous and heterologous molecular conformations, via processes such as the immune tolerance.

It is obvious that nowadays, the majority of association studies use preferentially SNPs as genetic markers, since their frequency in the genome is much higher and they are located at many more sites of interest than microsatellites. In fact, as displayed above, association studies focus on SNPs that reside not only in exons but also in regulatory regions, such as cis-elements (promoters and operators) and splicing sites. Furthermore, being easier and more robust to genotype, SNPs are automation friendly, allowing for massive interrogation in multiple candidate genes format and also in truly genomic scale studies, as it is the case in GWAS. However, the future of genetic analysis in autoimmune diseases and its corresponding applications in treatment, healthcare, and population studies lie in the rapid progress of next generation sequencing platforms which in the very next years will attain the sequencing of whole exome, transcriptome or genome, in a low-cost and accurate manner.

## Figures and Tables

**Table 1:** HLA alleles associated with MG in distinct subgroups of the disease and various ethnic populations.

MG subgroup	HLA locus	HLA allele or serological type	Ethnic group or descent	Number of cases	*P* value	Reference
*Thymoma-related MG *						

—	HLA-A	A*25	French	78	2.6 × 10^−3 ^	[23]
B2 type of thymoma	HLA-A	A*02^#^	French	27	4.13 × 10^−4^	[23]
—	HLA-DQA1	DQA1*0401	Northern Chinese	41	1.1 × 10^−2^	[24]
—	HLA-DQB1	DQB1*0604	Northern Chinese	41	1 × 10^−3^	[24]

*Nonthymomatous MG *						

Thymus hyperplasia	HLA-DRB1	DR3 DR7^#^	Caucasian	192	<1 × 10^−6^ <1 × 10^−6^	[9]
Thymus hyperplasia	HLA-DRB1	DR16	Caucasian	192	1.1 × 10^−5^	[9]
Thymus hyperplasia	HLA-DRB1	DR9	Caucasian	192	2 × 10^−3^	[9]
ATA (+) with no thymic abnormalities	HLA-DRB1	DR7	Caucasian	59	1 × 10^−2^ ^b^	[9]
ATA (−) with no thymic abnormalities	HLA-DRB1	DR3	Caucasian	60	2 × 10^−4^ ^b^	[9]
—	HLA-C	C*0701	Swedish	438	1.02 × 10^−3^	[25]
LOMG > 60 years	HLA-DRB1	DRB1*15 : 01	Norwegian	99	7.6 × 10^−5^ ^a^	[26]
EOMG < 40 years	HLA-B	B*08	Norwegian	154	2.5 × 10^−13^ ^a^	[26]
EOMG & MG with onset 41–59 years & LOMG	HLA-DRB1	DRB1*13 : 01^#^	Norwegian	339	4.71 × 10^−4^ ^a^	[26]
EOMG	HLA-DRB1	DRB1*08^#^ DRB1*09	Northern Han Chinese	52	7 × 10^−3^ 3.3 × 10^−2^	[27]
LOMG	HLA-DRB1	DRB1*07	Northern Han Chinese	39	3 × 10^−2^	[27]
Ocular MG	HLA-DRB1	DRB1*09 DRB1*12^#^	Northern Han Chinese	34	5.35 × 10^−5^ 2 × 10^−2^	[27]
AChR (+)	HLA-DRB1	DRB1*08^#^	Northern Han Chinese	44	2 × 10^−2^	[27]
AChR (−)	HLA-DRB1	DRB1*09	Northern Han Chinese	30	2 × 10^−3^	[27]
—	HLA-B	B*08	Saudi	109	3.4 × 10^−4^ ^a^	[28]
—	HLA-B	B*50^#^	Saudi	109	3.4 × 10^−3^ ^a^	[28]
—	HLA-DQA1	DQA1*0101/2	Iranian	104	—	[29]
—	HLA-DQB1	DQB1*0502	Iranian	104	—	[29]
Generalized EOMG	HLA-DRB1 & HLA-DQB1	DRB1*03 DQB1*02	Tunisian	30	<10^−3^ ^a^ <10^−3^ ^a^	[30]
Generalized LOMG	HLA-DRB1 & HLA-DQB1	DRB1*04 DQB1*0302	Tunisian	18	1.3 × 10^−3^ ^a^ <10 ^−3^ ^a^	[30]
Generalized MG/male patients	HLA-DRB1 & HLA-DQB1	DRB1*03 DQB1*02	Tunisian	26	<10 ^−3^ ^a^ <10 ^−3^ ^a^	[30]
Generalized MG/female patients	HLA-DRB1 & HLA-DQB1	DRB1*04 DQB1*0302	Tunisian	22	<10^−3^ ^a^ 1.6 × 10^−3^ ^a^	[30]
EOMG with thymus hyperplasia	HLA-B	B*08	North European	649	2.87 × 10^−113^ ^a^	[19]
EOMG with thymus hyperplasia	HLA-DRB1	DRB1*07^#^	North European	649	3.2 × 10^−8^ ^a^	[19]
EOMG with thymus hyperplasia	HLA-DRB1	DRB1*16	North European	649	1.3 × 10^−11^ ^a^	[19]

*MuSK-MG *						

—	HLA-DRB1 & HLA-DQB1	DR14-DQ5 haplotype	Dutch	23	4.9 × 10^−5^ ^a^	[31]
—	HLA-DQB1	DQB1*0502	Italian	37	7.3 × 10^−6^ ^a^	[32]
	HLA-DRB1	DRB1*16	Italian	37	2.3 × 10^−9^ ^a^	[32]

^#^Negative association implying a protective role of the specific allele in MG.

^a^Adjusted according to Bonferroni correction for multiple comparisons.

^b^ *P* value calculation was based on the comparison between ATA (+) and ATA (−) MG patients.

**Table 2:** Association of *PTPN22* rs2476601 and rs2488457 SNPs with distinct MG subgroups, in six different populations.

SNP/Associated allele	Ethnic group or descent	MG subgroup	Number of cases	*P* value	Reference
rs2476601/1858T	North European	EOMG with thymus hyperplasia	649	8.2 × 10^−10^ ^a^	[19]
	French	Nonthymoma & ATA (−)	293	5.9 × 10^−4^	[21]
		Nonthymoma & ATA (+)	97	NS	
		Thymoma	80	NS	
	Swedish Caucasian	All patients	409	2.7 × 10^−4^	[20]
		Thymus hyperplasia	142	1.4 × 10^−4^	
		AChR (+)	350	4.9 × 10^−4^	
		Thymus hyperplasia & AChR (+)	128	6.6 × 10^−5^	
	German & Hungarian	All nonthymoma MG patients	208	NS	[45]
		Nonthymoma & ATA (+)	50	7 × 10^−3^ ^a^	
		Nonthymoma & ATA (−)	152	NS	
		AChR (+)	164	<0.05	
		Thymoma	33	NS	
	German Caucasian	Thymoma	79	2.8 × 10^−3^	[46]
		EOMG	—	3.4 × 10^−4^	
	Italian Caucasian	All MG patients	356	NS^b^	[47]
		EOMG	117	NS^b^	
		AChR (+)	252	NS^b^	
		MuSK (+)	72	NS^b^	
		Thymoma	56	NS^b^	
		Nonthymoma	300	NS^b^	
	Meta-analysis of the [20, 21, 45, 46]	All MG patients	—	<1 × 10^−5^	[47]
		Nonthymoma	—	<1 × 10^−5^	
		AChR (+)	—	<1 × 10^−5^	
		Thymoma	—	NS	

rs2488457/-1123C	Italian Caucasian	AChR (+) with low titer ≤ 10 nM	50	0.019	[47]

^a^Adjusted according to Bonferroni correction for multiple testing.

^b^ *P* values were calculated based on genotype frequencies between patients and controls.
